# Cholesterol-Inulin Conjugates for Efficient SN38 Nuclear Delivery: Nanomedicines for Precision Cancer Therapy

**DOI:** 10.3390/cancers14194857

**Published:** 2022-10-04

**Authors:** Nicolò Mauro, Mara Andrea Utzeri, Roberta Cillari, Cinzia Scialabba, Gaetano Giammona, Gennara Cavallaro

**Affiliations:** 1 Laboratory of Biocompatible Polymers, Department of Scienze e Tecnologie Biologiche Chimiche e Farmaceutiche (STEBICEF), University of Palermo, Via Archirafi 32, 90133 Palermo, Italy; 2 Advanced Technology Environment Network Center, Viale Delle Scienze Ed. 18, 90128 Palermo, Italy

**Keywords:** inulin, SN38, drug delivery, polymeric micelles, colorectal cancer, triple negative breast cancer

## Abstract

**Simple Summary:**

Severe limitations of conventional chemotherapy including unspecific biodistribution both at the tissue and cell organelle level have led to the necessity of developing precise and personalized therapeutic strategies. Accordingly, nanomedicine has aroused increased attention due to the versatility and precision of nano-sized drug delivery systems. One of the main advantage offered by well-designed nanoplatforms is the possibility to load, stabilize, and deliver (also at intracellular level) hydrophobic anticancer drugs, whose clinical use is strongly affected by their lower bioavailability. In this overview, the synthesis of polymeric nano-sized core-shell micelle-like nanostructures is a promising delivery strategy for hydrophobic drugs due to their excellent drug loading efficacy, stability in aqueous media, and versatile design. On these grounds, we developed stable and biodegradable inulin-based micelles for the delivery of SN38, chosen as a powerful poorly water-soluble anticancer drug. We designed an amphiphilic polysaccharide hydrophobized by partial functionalization with thiocholesterol moieties (INU-Cys-TC) via disulfide redox-sensitive bridges. We demonstrated that INU-Cys-TC can self-assemble into micelles at low concentration, encapsulating a high amount of SN38 (INU-Cys-TC@SN38) to be released in a sustained fashion. INU-Cys-TC@SN38 has proven to be capable of entering cancer and normal cells, releasing their payload, especially inside cell nuclei, where SN38 can act, providing the maximum inhibition of its molecular target. However, due to a different cell localization, INU-Cys-TC@SN38 was much more toxic for cancer cells, improving the SN38 selectivity, precision, and enhancing its anticancer effect not only toward colorectal cells, but also for breast cancer cells. This is a good example of drug repurposing due to innovative environment-sensitive delivery strategies.

**Abstract:**

An amphiphilic inulin-thiocholesterol conjugate (INU-Cys-TC) was strategically designed as a biodegradable core-shell nanocarrier of 7-ethyl-10-hydroxy-camptothecin (SN38) to enhance its solubility and stability in aqueous media, thus exploiting its brilliant anticancer effect. INU-Cys-TC was designed to have the hydrophilic inulin backbone (external shell) partially functionalized with hydrophobic thiocholesterol moieties (internal core) through a biodegradable disulfide bond due to cysteamine bridges. Thiocholesterol moieties impair redox-sensitive self-assembling abilities, yielding to nano-sized micelles in aqueous media capable of efficiently encapsulating a high amount of SN38 (DL = 8.1%). Micelles (INU-Cys-TC@SN38) were widely characterized, demonstrating an effective and stable delivery strategy to overcome the poor water-solubility of SN38. SN38-loaded micelles showed a gradual and prolonged release of SN38 over time, and a cell- and time-dependent cytotoxicity. In particular, we show that micelles efficiently deliver SN38 inside cell nuclei, and, compared to normal cell lines, they can also enter cancer cells by endo-lysosomes, where a complete degradation can occur releasing the drug payload. Overall, the proposed micelles appear potentially effective as nanomedicines for precision cancer therapies of colorectal and breast cancer, thus improving the SN38 therapeutic index and extending its use in a huge plethora of cancers.

## 1. Introduction

Nanomedicines aim to overcome limitations of unspecific conventional chemotherapy (e.g., dose-limiting toxicity, severe side effects, multidrug resistance-MDR), exploiting the unique abilities of nanocarriers of modulating the pharmacokinetic and pharmacodynamic profiles of drugs, and enhancing their therapeutic index in order to improve the patients’ quality of life [1,2]. In recent decades, many works and pre-clinical studies have focused their attention on the development of nano-sized polymeric micelles as a powerful therapeutic strategy to surmount drug administration challenges, with particular attention to the delivery of hydrophobic drugs [3,4]. Indeed, many anticancer drugs display potent in vitro effects, but have also poor in vivo efficacy due to unsatisfactory bioavailability and intracellular localization [5,6]. Furthermore, many clinical trials fail because of the poor bioavailability of water-insoluble drugs, which is ascribed to almost half of the 150,000 new drugs synthesized annually by pharmaceutical companies, and it is also claimed to reduce the performance of more than 10% of successfully marketed drugs [7]. Polymeric core-shell micelles encapsulating drugs into the inner hydrophobic core guarantee higher stability, long circulation, and accumulation at the desired therapeutic concentration in the site of action, maximizing their pharmacological effects [8,9]. Furthermore, in order to improve the survival rate of cancer patients by overcoming the side effects toward off-target tissues, a possible strategy is the targeted delivery of anticancer drugs specifically to the intracellular compartment where they can produce effects (usually nuclei). Thus, the use of drug delivery systems such as polymeric micelles, which improve cell uptake and allow anticancer drug accumulation in peri-nuclear and nuclear regions, is highly recommended. Moreover, the facile preparation of nano-micelles by self-assembling in physiological conditions makes them an appealing, simple, and scalable formulation in drug delivery [10,11]. In order to obtain a suitable balance of loading, stability, systemic circulation, and delivery to the target tissue, several researchers have explored different strategic combinations of hydrophilic polymer and hydrophobic moieties, which constitute the outer shell and the inner core of the micelle structure, respectively [12,13]. The use of core-shell micelles with a diameter lower than 300 nm usually leads to tumor accumulation by the enhanced permeability retention (EPR) effect after systemic administration, and thus improves the local distribution of poorly water-soluble drugs in the tumor microenvironment, minimizing off-target effects [14]. 

Among the plethora of biomaterials useful to produce micelle-like nanostructures, inulin has aroused increasing attention due to its advantageous properties such as low molecular weight (~5000 Da), hydrophilicity, biodegradability, high biocompatibility, and also the possibility of being easily functionalized in the side chain, obtaining derivatives with different potentialities [15,16,17]. From a chemical standpoint, inulin consists of a linear chain of glucopyranose end-capped (β-1,2) fructose repeating units carrying a high amount of secondary and primary hydroxyl functional groups suitable for further functionalization [18,19]. The structural flexibility of inulin makes it an appealing and versatile biomaterial for different applications.

Therefore, the synthesis of highly hydrophobized inulin derivatives by partial substitution of hydroxyl groups with hydrophobic moieties represents an interesting strategy to obtain amphiphilic polysaccharide derivatives with self-assembling capacity, useful for the delivery of hydrophobic anticancer drugs [19,20].

Among them, 7-ethyl-10-hydroxy-camptothecin, also known as SN38, represents a hydrophobic anticancer drug that captures the scientific interest due to its powerful anticancer activity versus multiple cancers [21]. It is the biologically active metabolite of irinotecan, a topoisomerase I inhibitor, which is up to 20,000 times more potent than irinotecan, thus achieving a therapeutic effect at a lower dose [22]. The direct use of the active metabolite SN38 offers the advantage of avoiding hepatic metabolism by carboxylesterases, which is highly affected by the interpatient variability of the enzymatic activity, reducing associated severe side effects (e.g., myelosuppression, acute cholinergic-like syndrome, neutropenia), and minimizing the variability of the SN38 level [23]. However, the clinical use of SN38 is highly affected by its low solubility in water (<5 μg mL^−1^) and its instability in neutral or alkaline conditions, which induces the conversion from the active closed-form lactone ring to the inactive open-ring of carboxylate form [24]. This precludes its systemic administration and effective accumulation in cancer cell nuclei, thus reflecting in a poor efficacy in vivo. Therefore, the development of a functional formulation of SN38, which is able to enhance its water-solubility and simultaneously protects the active lactone ring, poses a great challenge for the scientific community [25].

On these grounds, the focus goal of the work was to develop a stable core-shell nanostructure for the efficient delivery of SN38. To fulfill this purpose, an amphiphilic inulin-derivative was rationally designed starting from a backbone of inulin successively functionalized in two steps with cysteamine and thiocholesterol moieties. The cysteamine spacer was selected because of its biodegradability, being an endogenous compound produced by different tissues, and for its ability to form bio-reducible and reversible bonds with a huge range of thiolated hydrophobic compounds. This will provide biodegradable macromolecular amphiphiles able to self-assemble into redox-sensitive micelles [26,27]. Thiocholesterol was chosen as an endogenous biocompatible side chain linked by S–S bonds and it is suitable to make inulin partially and reversibly hydrophobic. This bioconjugate is expected to load SN38 inside micelles to release their payload in situ by the high level of reduced glutathione usually observed inside cancer cells, thus improving its selectivity toward tumors and minimizing the side effects toward healthy cells [26,28,29,30]. 

The obtained inulin-graft copolymer, named INU-Cys-TC, was designed to reversibly self-assemble into micelles in water at low concentration and to efficiently entrap a high amount of SN38. We studied how SN38-loaded micelles overcome limitations due to the reduced water-solubility of SN38, reaching a high concentration in cancer cell nucleiand exploiting its anticancer activity toward different kind of cancer cells, such as breast cancer and colorectal cancer cells. We believe that the present work represents a good example of drug repurposing due to innovative targeted delivery strategies.

## 2. Materials and Methods

Inulin from dahlia tubers, N,N-dimethylformamide (DMF, >99.9%), bis-(4-nitrophenyl) carbonate (BNPC, 99.5%), cystamine di-hydrochloride (98.5%), dithiothreitol (DTT, 99%), thiocholesterol (TC, 99.5%), hydrogen peroxide (30% *v*/*v*), pyrene (99.5%), lithium bromide (99.5%), Human Serum Albumin (HSA), PBS, and SpectraPor^®^ Float-A-Lyzer^®^G2 (100–500 Da) were purchased from Sigma Aldrich (Milan, Italy) and used as received. Triethylamine (TEA, 99.5%) was purchased from VWR International (Milan, Italy). Potassium iodide (99.5%) was purchased from CARLO ERBA Reagents (Milan, Italy). SN38 (99.5%) was purchased from abcr GmbH (Karlsruhe, Germany). LysoTracker™ Red DND-99 and 4’, 6’-diamidino-2-phenylindole (DAPI) were purchased form Thermo-Fischer Scientific (Milan, Italy).

Dulbecco’s minimum Essential medium (DMEM), fetal bovine serum (FBS), L-glutamine, penicillin, streptomycin, and amphotericin B were purchased from EuroClone (Milan, Italy). The CellTiter 96^®^ AQueous One Solution Cell Proliferation Assay (MTS) was purchased from Promega (Milan, Italy).

Distortionless Enhancement by Polarization Transfer (DEPT), ^13^C NMR, and ^1^H NMR spectra were recorded using a Bruker 100 MHz and 300 MHz instrument, respectively.

The average weighted molecular weight (Mw) and polydispersity (PD) of INU-Cys-TC and the parent precursor INU-Cys-SH were evaluated by size exclusion chromatography (SEC). SEC analysis was performed with an Agilent 1260 Infinity instrument equipped with a Phenogel™ column connected in series to right angle (RA) and low angle (LA) light scattering (LS) detectors, and a refractive index (RI) detector. The mobile phase was a 0.01 M lithium bromide in DMF (flow rate 0.8 mL min^−1^).

### 2.1. Synthesis of Cysteamine-Functionalized Inulin (INU-Cys-SH)

Inulin (M_W_ 5 kDa, 375 mg, 0.075 mmol) was dispersed in 6 mL of N,N-dimethylformamide (DMF) and a solution of bis-(4-nitrophenyl) carbonate (BNPC, 354 mg, 1.16 mmol) in DMF (1.5 mL) was added dropwise with stirring. The mixture was conducted under microwave at 25 W and 60 °C for 1 h using a CEM Discover Microwave Reactor. The obtained product was added to a mixture of cystamine di-hydrochloride (157.19 mg, 0.69 mmol) and triethylamine (TEA) (2.1 mmol) in DMF (3 mL), and stirred for 2 h. The product was then crystalized in dichloromethane/diethyl ether (DCM/Et_2_O) 33:67 (70 mL). The product was retrieved by centrifugation (12,000 rpm, 15 min), and suspended in 10 mL of ultrapure water. After that, the pH was adjusted to 5, and then dithiothreitol (DTT) (1.077 g, 6.9 mmol) was added. The product was purified by dialysis versus water at pH 5 using a SpectraPor^®^ Float-A-Lyzer^®^G2 (100–500 Da), filtered on paper and freeze-dried to yield to a white powder (INU-Cys-SH) with a 60% yield.

^1^H-NMR (DMF-d7, 300 MHz): δ 2.67 ppm (2H_a_, -NH-CH_2_-CH_2_-S- Cys), 3.29 ppm (2H_b_, -NH-CH_2_-CH_2_-S- Cys), 3.73 ppm (H_c_,H_d_,H_g_, -CH-CH_2_-OH INU), 4.08 ppm (H_e_, -CH-OH INU), 4.27 ppm (H_f_, -CH-OH INU), 4.80 ppm (OH_h,_ INU), 4.92 ppm (OHi, INU), 5.39 ppm (OH_l_, INU), 8.03 ppm (DMF).

### 2.2. Synthesis of Inulin-Graft-Thiocholesterol Conjugate (INU-Cys-TC)

INU-Cys-SH (50 mg) was solubilized in anhydrous DMF (3 mL) and a solution of thiocholesterol (TC) in DMF (32.22 mg, 0.08 mmol, 2 mL) was slowly added dropwise under vigorous stirring. The mixture was heated, obtaining a limpid solution, then potassium iodide (13.28 mg, 0.08 mmol) and hydrogen peroxide 30% *v*/*v* (9.07 μL, 0.08 mmol) were added. The reaction was kept under a nitrogen atmosphere and with stirring overnight. The product was purified by dialysis versus water using a SpectraPor^®^ Float-A-Lyzer^®^G2 (100–500 Da) for 72 h. Successively, the product was filtered with 5 µm and 1.2 µm syringe filters before freeze-drying. A white powder, named INU-Cys-TC, was obtained with a 56% yield.

^1^H-NMR (DMF-d7, 300 MHz): δ 0.70 ppm (3H_15_, -CH_3_ TC), 0.87 ppm (3H_23_, 3H_24_, -CH_3_ - TC), 0.95 pmm (3H_18_, -CH_3_ - TC), 1.01 ppm (3H_16_, -CH_3_ TC), 1.05–1.20 ppm (axial H_1_, 2H_14_, 2H_11_, H_8_- TC), 1.28 ppm (2H_19_ and 2H_21_ - TC), 1.33–1.45 ppm (1H_7_ and 2H_20_-TC), 1.46–1.64 ppm (axial H_2_, axial H_9_, equatorial H_1_, 2H_10_, H_17_-TC), 1.78–2.07 ppm (equatorial H_2_, equatorial H_9_, 2H_12_, 2H_13_, H_22_ - TC), 2.16 ppm (H_3_, -S-CH-CH_2_CH_2_- TC), 2.31 ppm (2H_6_, -C=CH-CH_2_- of TC), 2.38 ppm (2H_4_, -S-CH_2_-CH_2_-CCHCH_2_- of TC), 2.57–2.70 ppm (2H_a_, -NH-CH_2_-CH_2_-S- Cys), 3.29 ppm (2H_b_, -NH-CH_2_-CH_2_-s- Cys), 3.73 ppm (H_c_,H_d_,H_g_, -CH-CH_2_-OH INU), 4.08 ppm (H_e_, -CH-OH INU), 4.27 ppm (H_f_, -CH-OH INU), 4.49–4.55 ppm (partial oxidation of INU end-chain), 4.83 ppm (OH_h_, INU), 4.97 ppm (OHi, INU), 5.41 ppm (OH_l_, INU), 5.83 ppm (H_5_, -C=CH-, TC).

^13^C-NMR (DMF-d7, 300 MHz): δ 12.2 ppm (C_16_, -CH_3 TC_), 19.2 ppm (C_19_, -CH_3 TC_), 19.7 ppm (C_17_, -CH_3 TC_), 21.6 ppm (C_10 TC_), 22.9 ppm (C_24_, -CH_3 TC_), 23.1 ppm (C_25_, -CH_3 TC_), 24.4 ppm (C_21 TC_), 24.8 ppm (C_13_-C_14 TC_), 28.6 ppm (C_23 TC_), 28.9 ppm (C_2 TC_ -C_a’ Cys_), 30.5 ppm (m, DMF), 32.4 ppm (C_1_-C_7 TC_), 32.6 ppm (C_8 TC_), 34.8 ppm (C_4 TC_), 35.7 ppm (m, DMF), 36.5 ppm (C_18 TC_), 36.9 ppm (C_20 TC_), 39.9 ppm (C_3 TC_), 40.1 ppm (C_a Cys_), 40.5 ppm (C_11_), 40.6 ppm (C_b Cys_), 44.8 ppm (C_b’ Cys_), 51.0 ppm (C_9_), 56.9 ppm (C_15_), 57.4 ppm (C_12_), 62.6 ppm (C_c INU_), 63.0 ppm (C_g INU_), 64.6 ppm (partial oxidation of INU end-chain), 73.8 ppm (C_n INU_), 75.7 ppm (C_e_, C_f_, C_o INU_), 78.9 ppm (C_p INU_), 83.4 ppm (C_d_, C_m INU_), 100.6 ppm (C_5_, -C=C- _TC_), 121.3 ppm (C_6_, -C=C- _TC_).

### 2.3. Determination of the Critical Aggregation Concentration (CAC) of INU-Cys-TC

The critical aggregation concentration (CAC) of INU-Cys-TC was determined by spectrofluorometric analysis using pyrene as hydrophobic fluorescence probe. In particular, 5 μL of pyrene’s solution in acetone (6 × 10^−5^ M) were placed in Eppendorf^®^ tubes and after the complete evaporation of acetone, 500 μL of the water dispersion of INU-Cys-TC (9.75 × 10^−5^ mg mL^−1^–1.00 mg mL^−1^) were added and incubated overnight. The emission spectra of the samples under excitation at 333 nm were recorded using a spectrofluorometer (Jasco FP-8500). The 373 nm (I_1_)/384 nm (I_3_) emission intensity ratio was evaluated to calculate the concentration of INU-Cys-TC corresponding to the formation of polymeric aggregates in aqueous dispersion.

### 2.4. Atomic Force Microscopy (AFM) of INU-Cys-TC

Atomic force microscopy (NTEGRA PRIMA AFM by NT-MDT Spectrum Instruments, Großhansdorf, Germany) was used to evaluate the size distribution of INU-Cys-TC micelles. Samples (0.1 mg L^−1^, 20 μL) were deposited on the MICA substrate and dried in vacuum (10 mbar). The analysis was performed in non-contact modality, using a triangular probe (resonance frequency = 1400 kHz, tip radius = 5 nm). AFM images were processed by using Gwyddion Software. 

### 2.5. Scanning Electron Microscopy (SEM) of INU-Cys-TC

Scanning electron microscopy was performed by a ESEM Philips XL30 operating at 30 kV to evaluate the morphological characteristics of INU-Cys-TC aggregates. Samples were prepared by depositing an aqueous dispersion of aggregates filtered on 0.45 μm (15 μL, 0.1 mg mL^−1^) on a carbon dish. All samples were sputtered with gold nanoparticles of about 8 nm of diameter before performing observations. 

### 2.6. Preparation of SN38-Loaded INU-Cys-TC Micelles (INU-Cys-TC@SN38)

INU-Cys-TC@SN38 was prepared via the kneading method. Briefly, powders of both INU-Cys-TC (10 mg) and SN38 (2 mg) were placed in a mortar and pounded to obtain a homogeneous solid mixture. Powders were then wetted with ethanol (1 mL × 5) and kneaded until the almost complete evaporation of the solvent. The thin film obtained was then suspended in ultrapure water (7 mL) and stirred for 1 h. The suspension was filtered by 5 µm and 0.45 µm syringe filters and freeze-dried (Labconco Freezone 6), obtaining a white powder named INU-Cys-TC@SN38 (Yield 67% *w*/*w*). 

### 2.7. Drug Loading Determination of INU-Cys-TC@SN38

The amount of SN38 loaded in INU-Cys-TC polymeric micelles was evaluated via HPLC analysis using an Agilent 1260 infinity HPLC System equipped with a C6-Phenyl column (Phenomenex^®^, Torrance, CA, USA) and a mobile phase consisting of a mixture of acetonitrile/phosphate buffer pH = 3 (60:40), operating with a flow of 0.8 mL min^−1^. The sample for the HPLC analysis was prepared by dissolving a weighted aliquot of INU-Cys-TC@SN38 in 500 μL of DMSO and sonicating for 2 h. The solution was then made up to the volume of 25 mL in a volumetric flask by adding the eluent solution. The analysis was performed in isocratic conditions and the absorbance of SN38 was detected at 265 nm (t_R_ = 6.7 min). The drug loading was calculated by comparing the sample’s peak area with a calibration curve obtained from the HPLC analysis of SN38 standard solutions at a concentration ranging from 1 × 10^−3^ mg mL^−1^ to 4 × 10^−2^ mg mL^−1^.

### 2.8. Dynamic Light Scattering (DLS) and Zeta-Potential Measurements

The size distribution of both INU-Cys-TC and INU-Cys-TC@SN38 was evaluated by Dynamic Light Scattering (DLS) measurements. Analyses were performed on 1 mL of a water dispersion of either INU-Cys-TC or INU-Cys-TC@SN38 (1 mg mL^−1^) using a Malvern Zetasizer NanoZS instrument equipped with a 632 nm laser with a fixed scattering angle of 173°. Z-average and PDI were obtained from the analysis of the correlograms. The same apparatus was used for zeta-potential analyses measurements at 25 °C on either INU-Cys-TC or INU-Cys-TC@SN38 (1 mg mL^−1^). Zeta-potential values were calculated from electrophoretic mobility using the Smoluchowski relationship (Malvern Instruments Zetasizer Software, Malvern, UK).

### 2.9. Cumulative Drug Release Study of INU-Cys-TC@SN38

The drug release kinetic was determined by the quantification of SN38 released from INU-Cys-TC over time, in PBS pH = 7.4 containing 3.5% (*w*/*v*) of Human Serum Albumin (HSA). In particular, the solution of HSA in PBS was used to prepare 6 mL solutions of either free SN38 or INU-Cys-TC@SN38 at an equivalent drug concentration (27 μg mL^−1^). These solutions were distributed in Eppendorf^®^ tubes in six aliquots of 1 mL. At scheduled time intervals (0, 0.5, 1, 8, 24 and 48 h), one aliquot of each experimental set was sacrificed and treated with 1 mL of methanol and sonicated (10 min), then centrifuged (10 min, 12,000 rcf) and the supernatant was withdrawn. The pellet was resuspended in 0.5 mL of DMSO, sonicated (15 min) and centrifuged (10 min, 12,000 rcf). The amount of SN38 in the supernatant was quantified spectrophotometrically by comparing the absorbance at 390 nm with a calibration curve obtained by plotting the absorbance at 390 nm as a function of the concentration of SN38 standard solutions ranging from 1 × 10^−3^ to 1 × 10^−1^ mg mL^−1^. Data were normalized by considering the MeOH/DMSO partition coefficient of free SN38.

### 2.10. Biological Characterization

#### 2.10.1. Cell Internalization Study of INU-Cys-TC@SN38

Cell uptake of INU-Cys-TC@SN38 was evaluated on two cancer cells lines, namely human colorectal cancer cells (HCT-116) and human breast cancer cells (MCF-7), and normal human bronchial epithelial cells (16-HBE) after 2 h, 6 h, and 24 h of incubation. First, cells were seeded in a 2-multiwell plate with a density of 2 × 10^4^ cells/well (2 mL/well) and cultured in Dulbecco’s modified Eagle medium (DMEM) for 24 h at 37 °C and 5% of CO_2_. Then, the culture medium was replaced with a INU-Cys-TC@SN38 dispersion in fresh DMEM (0.25 mg mL^−1^, 1 mL/well) and cells were re-incubated. Then, each well was washed three times with Dulbecco’s phosphate-buffered saline (DPBS) (1 mL × 3) and cells were fixed with 4% buffered formaldehyde solution (pH 7.4). After 10 min of incubation at 37 °C, each well was washed with DPBS (1 mL × 3) and acidic organelles were stained with LysoTracker™ Red DND-99 by re-incubating for an additional 1 h. Then, after washing each well with DPBS (1 mL × 3), cell nuclei were stained with 4’, 6’-diamidino-2-phenylindole (DAPI) for 10 min. Cell uptake images were recorded in brightfield and in three fluorescence channels (Dapi-457 nm, Fitc-516 nm, and Texasred-603 nm) with a multichannel fluorescence microscope Axio Cam MRm (Zeiss, Jena, Germany).

#### 2.10.2. In Vitro INU-Cys-TC and INU-Cys-TC@SN38 Cytotoxic Effects

In a first experimental set, the cytocompatibility of empty INU-Cys-TC micelles was evaluated on HCT-116, MCF-7 and 16-HBE by MTS assays. In detail, cells were seeded in a 96-multiwell plate with a density of 1.5 × 10^4^ cells/well (200 µL/well) and incubated for 24 h at 37 °C and 5% of CO_2_. Successively, the culture medium of each well was removed and replaced with INU-Cys-TC dispersions in fresh DMEM at different concentrations (1000–25 µg mL^−1^, 150 µL) for 24 h and 48 h. After the established time, each well was washed with DPBS three times and a MTS solution in fresh culture medium (20 µL of MTS and 100 µL of DMEM) was added to each well. After an additional 3h of incubation at 37 °C and 5% of CO_2_, the absorbance at 492 nm was measured by using a microplate reader (Multiskan, Thermo, UK). Untreated cells were used as control. The cytotoxic effect of INU-Cys-TC@SN38 was evaluated as described above. Briefly, cells were incubated with a dispersion of INU-Cys-TC@SN38 in DMEM at different micelle concentrations corresponding to an equal free drug molarity of 165–3.3 µM (150 µL per well) for 24 and 48 h. The same experimental set was performed with free drug used as the positive control. The pharmacological efficacy was measured as a reduction in cell viability by the MTS assay. Untreated cells were used as the negative control. All biological experiments were performed in triplicate and results are reported as mean values.

### 2.11. Statistical Data Analysis

Statistical analysis was performed by the two-tailed *t*-test in Origin 8.5 software package. Comparisons were considered statistically significant at *p* < 0.05 (*), *p* < 0.01 (**), and *p* < 0.001 (***).

## 3. Results and Discussion

### 3.1. Synthesis of the Amphiphilic Cholesterol-Grafted-Inulin Conjugate (INU-Cys-TC)

The amphiphilic cholesterol-grafted-inulin conjugate, henceforth named INU-Cys-TC, consists of a inulin backbone strategically grafted with hydrophobic moieties of cholesterol by means of bio-reducible disulfide bonds. Inulin (α-D-glucopyranosyl-[β-D-fructofuranosyl](n-1)-d-fructo furanoside), a linear polysaccharide of about 5 kDa consisting of glucopyranose end-capped fructose units (β-1,2), was chosen as the hydrophilic backbone due to its remarkable water solubility, bio-eliminability and reactive primary hydroxyl groups, exploitable for successive functionalization. Cysteamine was employed as a biocompatible sulfide linker providing a side group for disulfide coupling [26,28,31]. On the whole, the conjugate is designed to release all components after bio-reduction processes. 

**Figure 1:**
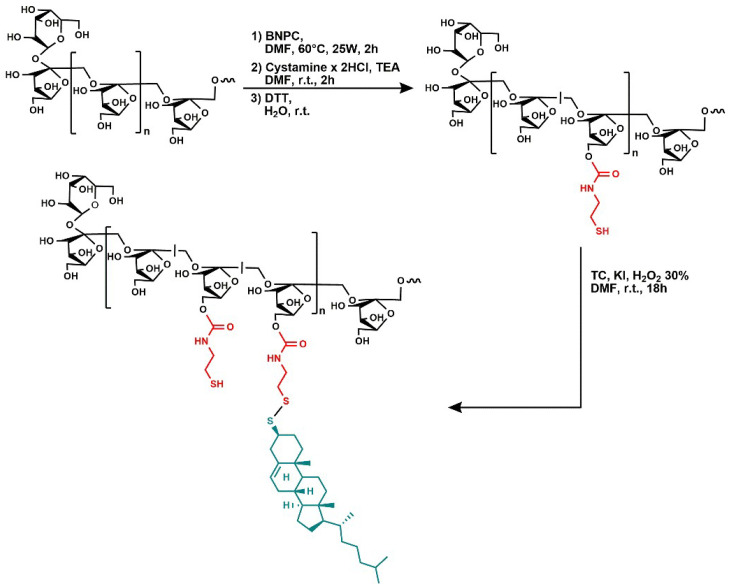
Schematic representation of the synthesis of INU-Cys-TC. In the first step, cytamine is first introduced as side pendants to be reduced, giving rise to cysteamine side groups. Finally, cholesterol groups were coupled by bio-reducible disulfide bonds.

INU-Cys-TC was synthetized by two reaction steps involving the functionalization of inulin with cysteamine and the successive conjugation with thiocholesterol (Figure 1). In the first step, inulin was partially functionalized with cysteamine, used as a linker to introduce reactive thiol pendants available for further functionalization by disulfide bond formation. The conjugation was performed by exploiting primary hydroxyl groups of inulin and amine end-groups of cystamine. Hydroxyl groups were activated by reacting with BNPC in an anhydrous DMF solution at 60 °C using a microwave reactor. The molar ratio between the activating agents and inulin repeating units of 0.5 and the microwave assisted synthesis allowed for a reproducible activation degree of hydroxyl groups (30% mol repeating units) to be achieved. Successively, activated hydroxyl groups of inulin underwent the nucleophile attack of amine groups of cystamine, considering a theoretical derivatization degree of 30%. To avoid crosslinking due to cystamine bridges, the activated polymer was added dropwise to excess amine functions under stirring. The intermediate, named Inu-Cys-NH_2_, was resuspended in water and an appropriate amount of dithiothreitol (DTT) was added to convert the cystamine lateral pendants to cysteamine side groups, obtaining the Inu-Cys-SH product.

The derivatization degree in the Cys side groups was evaluated by ^1^H-NMR (Figure S1) by comparing the integral of the peak at 2.65–3.30 ppm, relative to CH_2_ of the cysteamine portion, with that at 4.09–4.26 ppm of the glucopyranose portion. The integral ratio corresponded to 14 mol %, roughly equal to four cysteamine-containing repeating units for the inulin chain.

**Figure 2:**
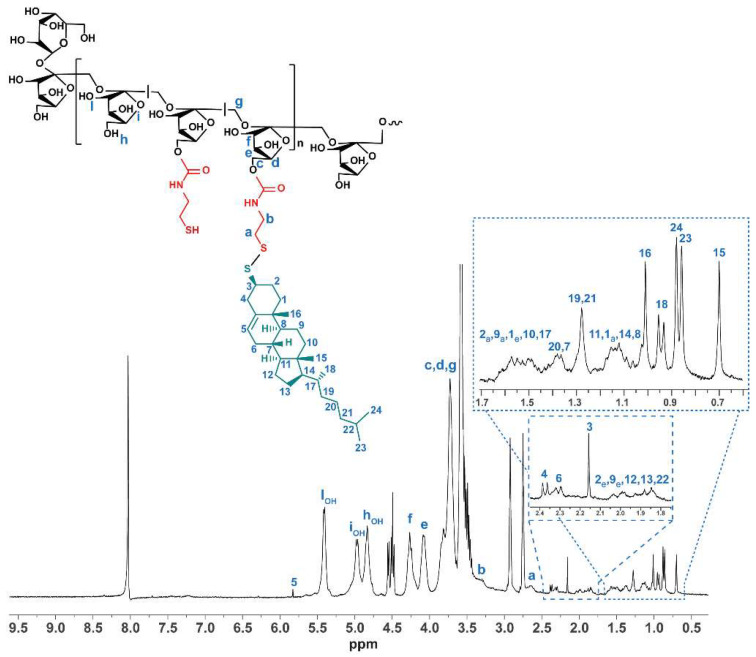
^1^H-NMR spectra of INU-Cys-TC (DMF-d_7_, 300 MHz). The structure and relative chemical shift assignments are reported as insertions.

Taking into account that Inu-Cys-SH is a modified hydrophilic polysaccharide unable to self-assemble into nanostructures that are useful to deliver highly hydrophobic drugs such as SN38, hydrophobic thiocholesterol moieties were introduced as side chains to the SH groups via S–S bridges to provide amphiphilic grafted copolymers able to form micelle-like nanostructures in water. Therefore, the second step involved the partial conjugation of thiocholesterol with cysteamine pendants by disulfide bonds in the presence of a strong oxidant agent such as hydrogen peroxide. As demonstrated by Ranucci et al., thiocholesterol linked by S–S bonds are stable in blood but cleavable inside cells, providing bio-eliminable and biocompatible lipophilic domains for the self-assembling of amphiphiles [26]. The obtained purified product, INU-Cys-TC, was characterized by ^1^H-NMR to evaluate the efficacy of the reaction scheme and the functionalization degree in TC moieties (Figure 2). The functionalization successfully occurred, as confirmed by the distinctive peaks of cholesterol in the range 0.5–2.5 ppm. In detail, marked peaks at 0.70–1.01 ppm, attributable to the five methyl groups of cholesterol, were considered to calculate the functionalization degree of INU-Cys-TC. The integral ratio cholesterol/glucopyranose portion corresponded to a derivation of 8% mol mol^−1^ of repeating units. The well-defined triplet at 4.48–5.51 ppm and doublet at 4.54–4.56 ppm could be attributed to the glucopyranose ring opening induced by the microwave assisted synthetic process.

**Figure 3:**
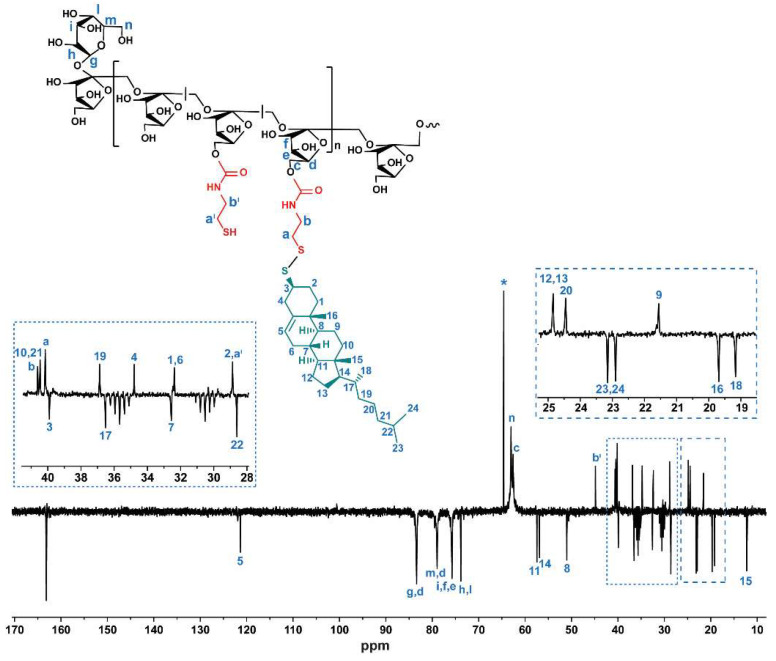
DEPT ^13^C-NMR of INU-Cys-TC (DMF-d_7_, 300 MHz). The structure and relative chemical shift assignments are reported as insertions.

Data obtained from ^1^H-NMR were further supported by ^13^C-NMR (Figure 3), which indeed presents both characteristic peaks of inulin (62.6–63.0 ppm relative to -CH-, 73.8–83.4 ppm relative to -CH_2_-) and typical peaks of thiocholesterol (12.2–23.1 ppm relative to methyl groups and 100.6–121.3 ppm relative to -C = CH-). In addition, peaks attributable to CH_2_ protons of cysteamine pendants (28.9 ppm and 44.8 ppm) are shifted to 40.1 ppm and 40.6 ppm, respectively, when side groups were bound to thiocholesterol by disulfide functions. According to literature [32], this shift is due to the oxidation of the thiol group adjacent to -CH_2_-, confirming the formation of disulfide bonds. Moreover, the peak signed at 64 ppm confirms the ring opening of the glucopyranose end-chain portion.

The average weighted molecular weight (Mw) and polydispersity (PD) of inulin derivatives were evaluated by SEC, and reported in Table 1. The Mw of Inu-Cys-SH and INU-Cys-TC were 3400 and 4400, respectively. Considering the starting Mw of inulin of about 5 kDa, the reduced Mw of Inu-Cys-SH of about 1700 Da revealed that the microwave assisted synthesis induced a partial hydrolysis of the polysaccharide chains. Conversely, the Mw increase observed after the conjugation with cholesterol (about 1000) is in agreement with the derivatization degree in TC moieties calculated for INU-Cys-TC (about 2–3 cholesterol moieties per chain, see NMR data), confirming the effectiveness of the synthetic procedure.

### 3.2. Physicochemical Characterization of INU-Cys-TC

**Figure 4:**
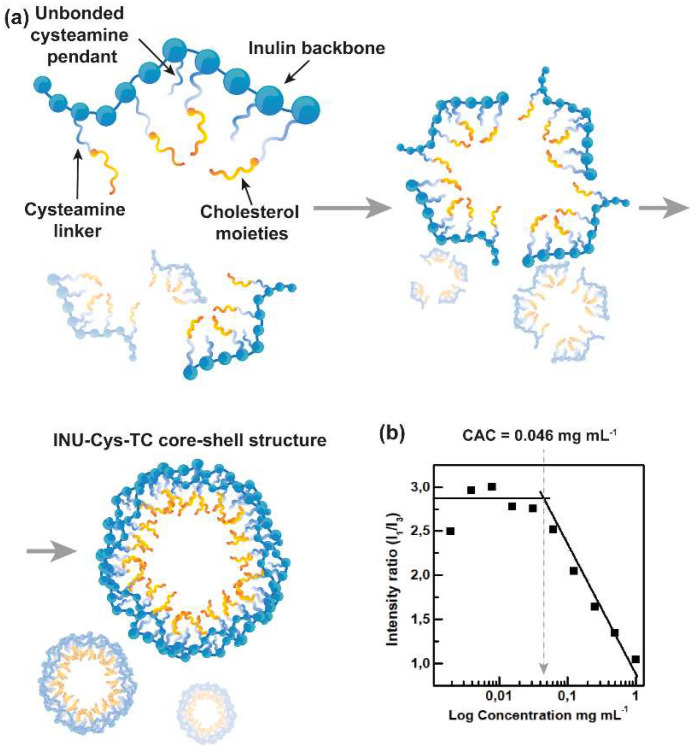
Representation of the self-assembling processes taking place during the formation of core-shell nanostructures (**a**). Pyrene assay analysis for CAC determination (**b**): the I_1_/I_3_ ratio obtained from the emission spectrum of pyrene usually changes when in the presence of hydrophobic pockets due to the self-assembling of amphiphilic polymers.

The ability of the amphiphilic INU-Cys-TC to self-assemble in water into stable polymeric core-shell nanostructures was investigated by the fluorescence pyrene assay using the latter as fluorescent probe (Figure 4a). The critical aggregation concentration (CAC) was calculated by plotting the I_373nm_/I_384nm_ obtained from the pyrene emission profile (**λ**_Ex_ 333 nm) in the presence of INU-Cys-TC as a function of the conjugate concentration. As shown in Figure 3b, the I_373nm_/I_384nm_ ratio decreased by increasing the concentration of the conjugate, suggesting that changes in the copolymer conformation occurred. Typically, the CAC value can be extrapolated from the intersection of the tangents to the sigmoidal curve, which for INU-Cys-TC corresponds to 0.046 mg mL^−1^ (Figure 4b). It is reasonable to believe that a such amphiphilic conjugate is a suitable candidate to deliver poorly soluble anticancer drugs such as SN38, thus providing an ideal concentration in physiological fluids, especially inside cancer cells upon cell internalization.

**Figure 5:**
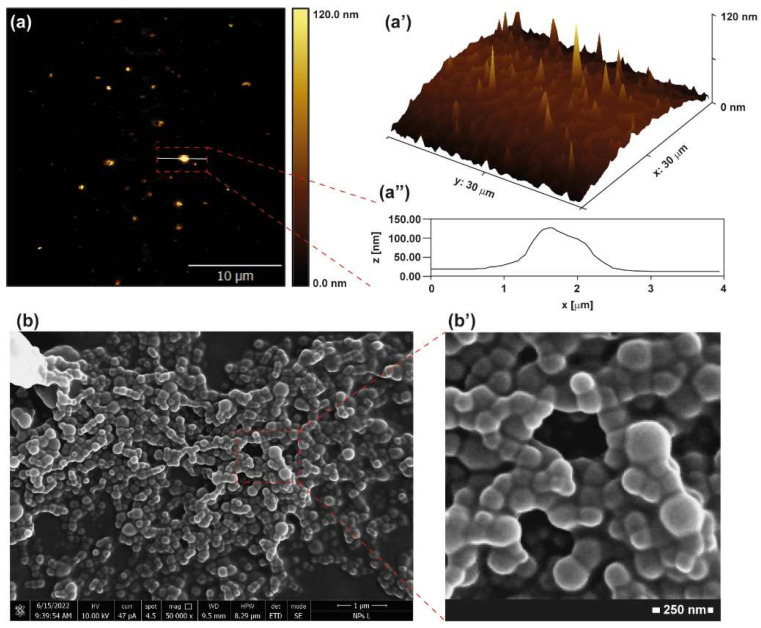
AFM images (**a**,**a’**): 3D AFM micrograph and heights’ profile of particles (**a’’**) were obtained from the rough data by Gwydion software. SEM images (**b**,**b’**).

Atomic force microscopy (AFM) micrographs of dried INU-Cys-TC core-shell structures show evidence of isolated spherical objects with a limited tendency to aggregate (Figure 5a). The average diameter extrapolated from the heights was about 120 nm (Figure 5a’–a’’) with a narrow size distribution. These homogeneous polymeric micelles seem suitable for efficient cellular internalization, as suggested by previously reported works where ellipsoid nanomedicines were able to efficiently enter cancer cells [33]. Furthermore, AFM data were confirmed by SEM analysis of dried INU-Cys-TC samples (Figure 5b,b’). In detail, SEM micrographs highlighted a homogeneous population of nanostructures with a spherical shape. This evidence was even more evident in the particular one reported in Figure 5b’, where core-shell structures were perfectly distinguishable from each other, confirming the lack of stable and strong aggregates.

### 3.3. Preparation and Characterization of Loaded INU-Cys-TC@SN38 Micelles

A moderate hydrophobization of the inulin backbone with thiocholesterol chains allows for obtaining an amphiphilic polysaccharide that is able to self-assemble in water into nano-sized micelles at a very low concentration. It might be expected that the hydrophobic core of micelles would provide a sufficient task to load poorly water-soluble drugs. With the aim of evaluating the ability of INU-Cys-TC micelles to act as a drug delivery system of hydrophobic drugs, they were loaded with 7-ethyl-10-hydroxy-camptothecin (SN38), the hydrophobic active metabolite of irinotecan, chosen as anticancer drug model. SN38 has aroused considerable attention in cancer therapy due to its improved anticancer activity if compared with the parent irinotecan. However, its clinical use is strongly limited by its aforementioned poor water-solubility and instability under physiological conditions [34]. Therefore, the encapsulation of SN38 into self-assembled micelles such as INU-Cys-TC could represent a valid delivery strategy to make it useful in cancer therapy. Furthermore, SN38 needs to reach the cell nuclei to provide the inhibition of topoisomerases. Hence, its encapsulation in a polysaccharide such as inulin provides a good chance to be internalized inside cancer cells, to be released at the peri-nuclear level and to diffuse throughout the cells including the nucleus [35,36]. 

INU-Cys-TC@SN38 micelles were prepared by kneading technique, starting from INU-Cys-TC and a theoretical drug loading of 20% *w*/*w*. The free drug was removed by sequential filtration, exploiting the reduced water solubility of SN38. This drug loading method, combined with the stable core-shell structures of INU-Cys-TC, allowed obtaining high drug loading levels of (8.1 ± 1.8% *w*/*w*), one of the highest ever reported to the best of our knowledge, with an encapsulation efficiency of about 41%. The high drug loading is of great importance to reach a therapeutic concentration at the right cell compartment (nuclei) after cell internalization, thus avoiding multidrug resistance (MDR) due to poor drug levels [6]. Indeed, it is commonplace that the inhibition of enzymes is a concentration-dependent mechanism that can be promoted only at relatively high local concentration of inhibitor molecules. As a role, an unsuitable local amount of drugs provokes MDR and thus recidivisms. 

### 3.4. Dynamic Light Scattering (DLS) Measurements

The hydrodynamic diameter and the polydispersity index (PDI) of empty and drug-loaded INU-Cys-TC micelles were measured on sample’s aqueous dispersions at a concentration above the CAC value (0.046 mg mL^−1^). As reported in Table 2, core-shell structures of INU-Cys-TC showed a hydrodynamic diameter of about 177 nm with a good polydispersity index, according to the size distribution data extrapolated from the AFM and SEM micrographs. However, after the encapsulation of SN38, a moderate increment in diameter occurred (~47 nm), attributable to strong hydrophobic interactions of SN38 with cholesterol moieties inside the core. In addition, samples displayed similar negative zeta-potential values, with a no relevant difference of about 7 mV. These data suggest the formation of well-structured polymeric micelles with a clear localization of SN38 molecules inside the hydrophobic core of assembled INU-Cys-TC conjugates. Additionally, DLS studies suggest that drug-loaded micelles have dimensions suitable for both systemic and oral administration, which would provide efficient biodistribution and effective cell internalization. In particular, even if nanosystems bigger than 10 nm are not able to enter cell nuclei, where SN38 usually acts, INU-Cys-TC conjugates are bio-reducible inside the cancer cells under the action of high intracellular level of glutathione. One can imagine that after the reduction of disulfide bonds inside cells, the hydrophilic inulin product will release the drug payload inside cancer cells, thus permitting the massive diffusion of SN38 inside the nuclei [26,37].

### 3.5. Cumulative Drug Release Study of INU-Cys-TC@SN38

**Figure 6:**
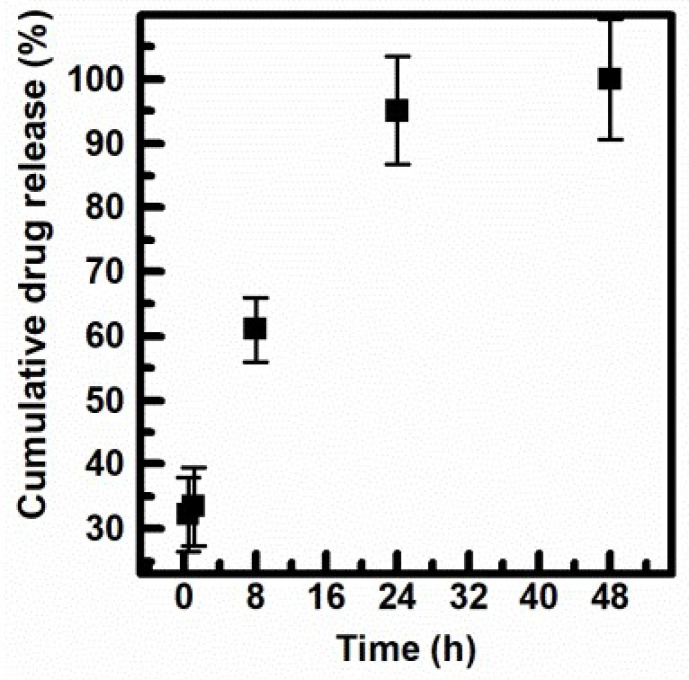
The cumulative drug release kinetic of INU-Cys-TC@SN38 in PBS pH 7.4 at 37 °C.

The drug release kinetic of INU-Cys-TC@SN38 micelles was studied at 37 °C in PBS pH = 7.4 solutions containing Human Serum Albumin (HSA) at the physiological blood concentration of 3.5% *w*/*v*. The amount of SN38 released at scheduled intervals of time was evaluated by precipitating both albumin and INU-Cys-TC in methanol and extracting the pellet of SN38 with DMSO. The concentration of the extracted SN38 in DMSO was quantified by HPLC and normalized by considering the MeOH/DMSO partition coefficient of the free SN38 previously evaluated. The obtained cumulative drug release is reported in Figure 6. It is self-evident that after the initial burst effect (32% release in 1 h), a slow and constant release of the drug payload occurred over time, reaching complete drug release over 32 h. It is interesting to note that the moderate burst effect observed is important to provide an initial available dose of free drug, which increases the concentration up to the therapeutic window. The sustained release observed up to 48 h maintained the right concentration over time. In addition, the sustained release of the drug payload confirmed the effectiveness of the core-shell nanostructure as a drug delivery system for a controlled and prolonged release mechanism. Indeed, after the administration of a nanomedicine (drug reservoir) with a diameter bigger than 5.5 nm (renal cut off), the clearance of the drug payload depends on the concentration of the free drug (released fraction) over time. Hence, the proposed micelles can potentially prolong blood circulation due to the slow drug release observed (100% release in 48 h). Furthermore, nanomedicines with diameters lower than 300 nm can accumulate inside solid tumors by the enhanced permeability retention (EPR) effect, thus impinging on the bioelimination processes and increasing the local drug bioavailability in situ [38,39].

### 3.6. Biological Characterization

#### 3.6.1. Cell Uptake Study of INU-Cys-TC@SN38

**Figure 7:**
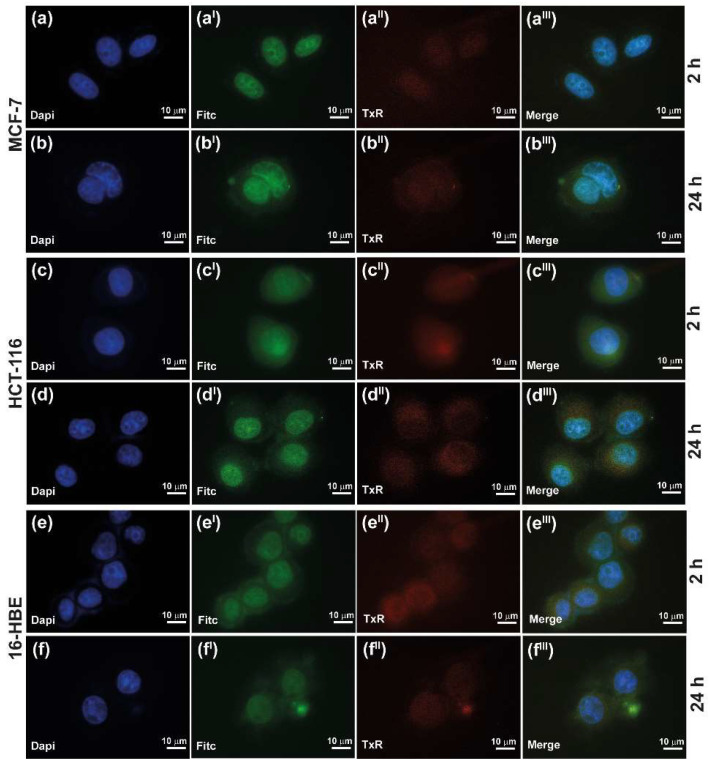
Cell uptake of loaded INU-Cys-TC@SN38 on MCF-7 (**a**–**a^III^**, **b**–**b^III^**), HCT-116 (**c**–**c^III^**, **d**–**d^III^**) and 16-HBE (**e**–**e^III^**, **f**–**f^III^**) after 2 h and 24 h of incubation. Lysosomes were stained with TxR (red), nuclei with DAPI (blue), and SN38 is self-stained (green).

The ability of the nanosystem to enter cancer cells through different cell membrane pathways was evaluated for INU-Cys-TC@SN38 micelles, exploiting the emission property of SN38 in the green region of the light spectrum. In particular, as reported elsewhere, SN38 is characterized by a remarkable emission in the green region (FITC channel), which represents an advantage to evaluate and monitor its cell internalization and localization by fluorescence microscopy and confocal analysis [40]. In order to better understand the main cell uptake pathway involved in the internalization of the delivered SN38 and its intracellular fate, lysosomes were stained with LysoTracker (TxR channel), while nuclei where stained with DAPI (blue channel). Multi-channel fluorescence micrographs highlighted that INU-Cys-TC@SN38 could efficiently enter all cell lines explored, both cancer and normal cells, since a marked green fluorescence was registered after only 2 h of incubation (Figure 7). Furthermore, SN38 is localized throughout cells (cytoplasm, lysosomes and nuclei) and especially inside nuclei, where SN38 acts as a topoisomerase inhibitor providing the main anticancer effect known for this drug. The fluorescence profile is comparable to that shown after 6 h (Figure S3) and 24 h (Figure 7), suggesting that a constant and high concentration of drug can be maintained in cancer cells, especially localized inside nuclei, due to the sustained release of INU-Cys-TC@SN38. This will guarantee suitable anticancer effects, avoiding MDR due to the low concentration of SN38 at the nuclear level. However, it is important to note that SN38 had a cytoplasmatic localization in MCF-7 only after 6 h (Figure S3) and 24 h (Figure 7b–b^III^) of incubation, while it appeared exclusively in the nuclei after 2 h of incubation (Figure 7a–a^III^). These could be attributed to a difference in the vesicle formation activity between the cell lines considered as well as to the internalization pathway adopted by micelles. Indeed, brightfield images (Figures S2–S4) of 16-HBE and HCT-116 confirmed a high density of vesicle already after 2 h, co-localized with green fluorescence of SN38 and in accordance with TxR images. It is also important to underline that inulin suffers from acid-dependent hydrolysis, which provokes the degradation of micelles inside lysosomes upon cell internalization. Therefore, the inulin-based nanocarrier could potentially disassemble in fructose and glucose inside the lysosome, favoring the release and diffusion of SN38 [41,42].

Overall, the uptake study conducted seems to suggest that INU-Cys-TC@SN38 core-shell nanostructures enter cancer and normal cells in a similar fashion, leading to a storage within lysosomes, which favored the degradation of the core-shell structure and the diffusion of the payload toward cytoplasm and nucleus. Moreover, the high density of SN38 inside nuclei confirms the effectiveness of the designed nanomicelles as carriers for specifically delivering drugs directly to the site of action, and keeps high intracellular concentrations suitable to combat resistant cancer cells.

#### 3.6.2. In Vitro Cytocompatibility and Anticancer Efficacy

**Figure 8:**
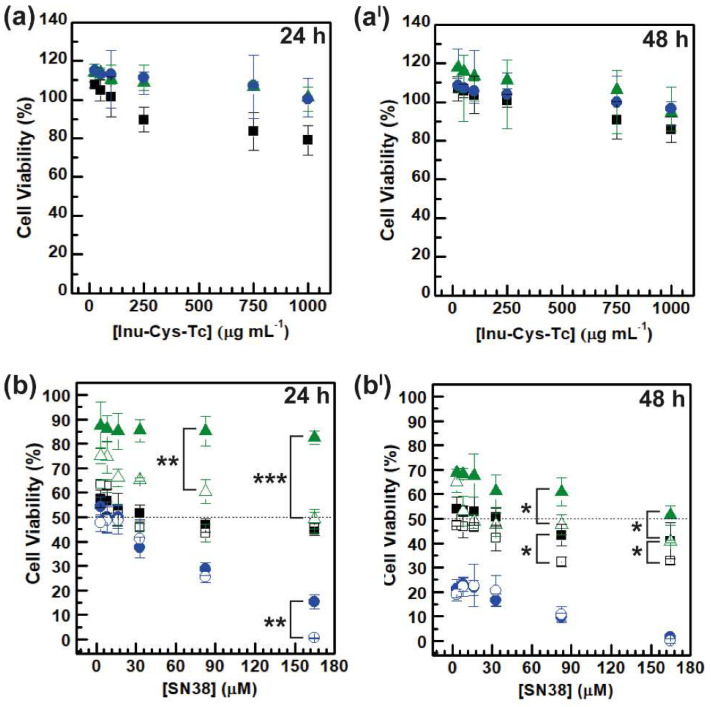
Study of the cytocompatibility of INU-Cys-TC (**a**,**a^I^**) and INU-Cys-TC@SN38 (**b**,**b^I^**) after 24 h (**a**,**b**) and 48 h (**a^I^**,**b^I^**) of incubation on MCF-7 (black), HCT-116 (blue), and 16-HBE (green). Full symbols correspond to INU-Cys-TC (**a**,**a^I^**) and INU-Cys-TC@SN38 (**b**,**b^I^**), while empty symbol correspond to free SN38 (**b**,**b^I^**). *p* < 0.05 (*), *p* < 0.01 (**), and *p* < 0.001 (***).

The cell viability of cells treated with INU-Cys-TC and INU-Cys-TC@SN38 was carried out on two cancer models, namely, breast cancer (MCF-7) and colorectal cancer (HCT-116), and one model of normal bronchial epithelial tissue (16-HBE) [43]. First, we evaluated the cytocompatibility of empty INU-Cys-TC micelles in a wide range of concentrations from 1000 to 25 μg mL^−1^. As displayed in Figure 8a,a^I^, INU-Cys-TC was highly cytocompatible (cell viability > 80–100%) on all tested cell lines in the entire range of concentrations considered as well as after 48 h of exposure. At the lower dosage tested (<0.25 mg mL^−1^) the conjugate enhanced the cell viability (cell viability > 100%) due to the typical proliferative behavior of the inulin backbone [35,44]. This is clear evidence that the proposed micelles are excellent candidates for drug delivery applications. 

On the other hand, to assess the cytotoxic effect of drug-loaded INU-Cys-TC@SN38 micelles, we assessed the cell viability using an equivalent amount of loaded micelles on the same cell lines. Hence, for comparative purposes, each concentration of INU-Cys-TC@SN38 was calculated by considering the drug loading previously calculated and data were reported as a function of the SN38 concentration (Figure 8b,b^I^). It was observed that there was a remarkable cell- and dose-dependent cytotoxic effect (Figure 8b,b^I^). After 24 h of incubation with INU-Cys-TC@SN38, both cancer cells underwent significant damage in a dose-dependent way. In particular, the anticancer effect was similar than that observed for free drug. However, this trend was not observed for healthy cells (16-HBE), where highly reduced cytotoxic effects were displayed in comparison to free SN38. In more detail, according to the well-known anticancer activity of SN38 versus colorectal tumor, INU-Cys-TC@SN38 induced up to the 100% of cell death after 48 h of incubation, even at the lower concentrations tested. Furthermore, INU-Cys-TC@SN38 also displayed a good anticancer effect on MCF-7, inducing 50% of cell death even at very low concentrations (50% cell viability at 30 μM).

This trend was much more evident considering the IC_50_ and I_max_ values calculated and reported in Table 3. The IC_50_ value calculated from the dose–response curve of HCT-116 confirmed the high effectiveness of SN38 when delivered by INU-Cys-TC@SN38 micelles since, surprisingly, it displayed the same IC_50_ of free SN38. Even the maximum inhibition level (I_max_) was comparable (I_max_ 99% vs. 100%). However, as expected, the cytotoxic effect induced on MCF-7 cells appeared ten-fold lower than that on HCT-116 (IC_50_ 33 mM and 3.3 mM, respectively), probably ascribable to the different degree of internalization by the vesicular pathway, as evidenced by the study of uptake. In fact, SN38 is a clinically relevant anticancer drug with selective efficacy for colorectal cancer. Furthermore, it is noteworthy that drug-loaded micelles had an IC_50_ value eight-fold higher than that obtained for the free drug on normal cells (16-HBE), suggesting a highly selective anticancer effect of SN38 when delivered at the nuclear level by the micelles. Additionally, the I_max_ values observed after 24 h and 48 h of incubation were lower for the drug-loaded micelles (18% vs. 55% after 24 h), implying a high cytocompatibility of INU-Cys-TC@SN38 on normal cells. The higher cytotoxicity observed on cancer cells can be due to the redox sensitivity of the disulfide bonds typically observed in cancer cells, where the cytoplasmatic concentration of reduced-glutathione is about ten-fold higher compared to normal cells [28,31]. This implies a fast degradation of the hydrophobic moieties linked by disulfide bridges (TC functions) inside lysosomes, and the consequent rapid diffusion of the drug payload, which induces fast and unreversible cell damages. 

On the whole, these data make INU-Cys-TC@SN38 an excellent core-shell nanostructure for the nuclear delivery of powerful hydrophobic anticancer drugs such as SN38 to overcome the poor water solubility, which limits its clinical use. Moreover, INU-Cys-TC@SN38 micelles, because of their high selectivity toward cancer cells and high drug loading, have a very huge potential as anticancer tools for the treatment of different kinds of cancers, among which are breast cancers, thus extending the use of SN38 not only as an anticancer drug for colorectal cancer.

## 4. Conclusions

In this work, we developed a biodegradable inulin-cholesterol derivative capable of forming core-shell nanostructures with micelle-like architecture, suitable for the delivery of highly hydrophobic anticancer drugs such as SN38. We proved that this peculiar biodegradable nanoplatform can be used for the treatment of many tumors. The architecture of the amphiphilic grafted conjugate INU-Cys-TC allows one to obtain self-assembling core-shell micelles (d = 220 nm) that are able to efficiently encapsulate a high amount of SN38 (8.1% *w*/*w*),one of the highest ever reported in the literature. The drug-loaded INU-Cys-TC@SN38 displayed promising anticancer effects on colorectal and breast cancer models, comparable to the use of free SN38 in vitro, but with the advantage of overcoming limitations such as its poor water solubility and the instability of the lactone ring al pH >6, which preclude extensive clinical applications for free SN38. 

We proved that INU-Cys-TC@SN38 micelles can enter cancer cells and normal cells, leading to an efficient and prolonged delivery of the payload inside nuclei, where SN38 can act as a topoisomerase inhibitor, fully providing its anticancer potential. This is particular evident in cancer cells, where micelles also co-localize with lysozymes, thus triggering biodegradation by disulfide cleavage and inulin hydrolysis. 

Compared to the free drug, loaded micelles improved the selectivity toward cancer cells. Indeed, the IC_50_ of micelles was eight-times higher in normal cells (16 HBE) with respect to the free drug. Therefore, the proposed biodegradable core-shell nanostructures represent an innovative strategy to overcome severe limitations in the clinical application of SN38, exploiting its well-known powerful anticancer activity, improving its selectivity for cancer cells, and repurposing its use in other severe malignant diseases such as breast cancer.

## Figures and Tables

**Table 1:** Derivatization degree (DD) calculated by ^1^H NMR, the average weighted molecular weight (Mw), and polydispersity (PD) evaluated by the SEC of inulin-derivatives.

Sample	DD_Cys_ (mol%)	DD_TC_ (mol%)	Mw	PD
INU-Cys-SH	14	-	3391	1.39
INU-Cys-TC	14	8	4413	1.53

**Table 2:** The DLS measurements of the zeta-average and zeta-potential of SN38-loaded and empty INU-Cys-TC micelles.

Sample	Z-Average (nm)	PDI	Zeta-Potential (mV)
INU-Cys-TC	177.7 ± 0.9	0.135	−25.3 ± 5.4
INU-Cys-TC@SN38	225.4 ± 0.7	0.136	−18.2 ± 3.9

**Table 3:** Calculated half minimal inhibitory concentration (IC_50_) and maximal inhibition (I_max_) of INU-Cys-TC@SN38 and free SN38 after 24 and 24 h of incubation on MCF-7, HCT-116, and 16-HBE.

Sample	Cell Line	IC_50_^24h^ (mM)	IC_50_^48h^ (mM)	I_max_^24h^ (%)	I_max_^48h^ (%)
*INU-Cys-TC@SN38*	MCF-7	47 ± 3.1	33 ± 1.4	55 ± 0.5	59 ± 0.7
	HCT-116	3.3 ± 0.3	<3.3	85 ± 0.6	99 ± 1.1
	16-HBE	>165	165 ± 9.0	18 ± 0.2	48 ± 0.8
*Free SN38*	MCF-7	17 ± 0.9	<3.3	55 ± 0.9	67 ± 0.9
	HCT-116	<3.3	<3.3	100 ± 0.3	100 ± 0.2
	16-HBE	130 ± 3.7	20 ± 0.6	50 ± 0.5	59 ± 0.3

## Data Availability

The data presented in this study are available on request from the corresponding author.

## Supplementary Materials

The following supporting information can be downloaded at: https://www.mdpi.com/article/10.3390/cancers14194857/s1, Figure S1: ^1^H-NMR spectrum of the intermediate Inu-Cys-SH; Figure S2: Cell uptake of loaded INU-Cys-TC@SN38 after 2 h of incubation.; Figure S3: Cell uptake of loaded INU-Cys-TC@SN38 after 6 h of incubation; Figure S4: Cell uptake of loaded INU-Cys-TC@SN38 after 24 h of incubation.
