# Supplementary material for: Cholesterol-Inulin Conjugates for Efficient SN38 Nuclear Delivery: Nanomedicines for Precision Cancer Therapy
Source: Cancers (Basel). 2022 Oct 4;14(19):4857. doi: 10.3390/cancers14194857 (PMC9563076; doi:10.3390/cancers14194857)
Supplement: Supplementary file 1 [file cancers-14-04857-s001.zip › cancers-1897958-supplementary.pdf]

Supplementary Materials

Cholesterol-Inulin Conjugates for Efficient SN38 Nuclear Delivery: Nanomedicines for Precision Cancer Therapy

Nicolò Mauro [1,*], Mara Andrea Utzeri [1], Roberta Cillari [1], Cinzia Scialabba [1], Gaetano Giammona [1] and Gennara Cavallaro [1,2]

1 Laboratory of Biocompatible Polymers, Department of "Scienze e Tecnologie Biologiche Chimiche e Farmaceutiche (STEBICEF), University of Palermo, Via Archirafi 32, 90123 Palermo, Italy

2 Advanced Technology Environment Network Center, Viale Delle Scienze Ed. 18, 90128 Palermo, Italy

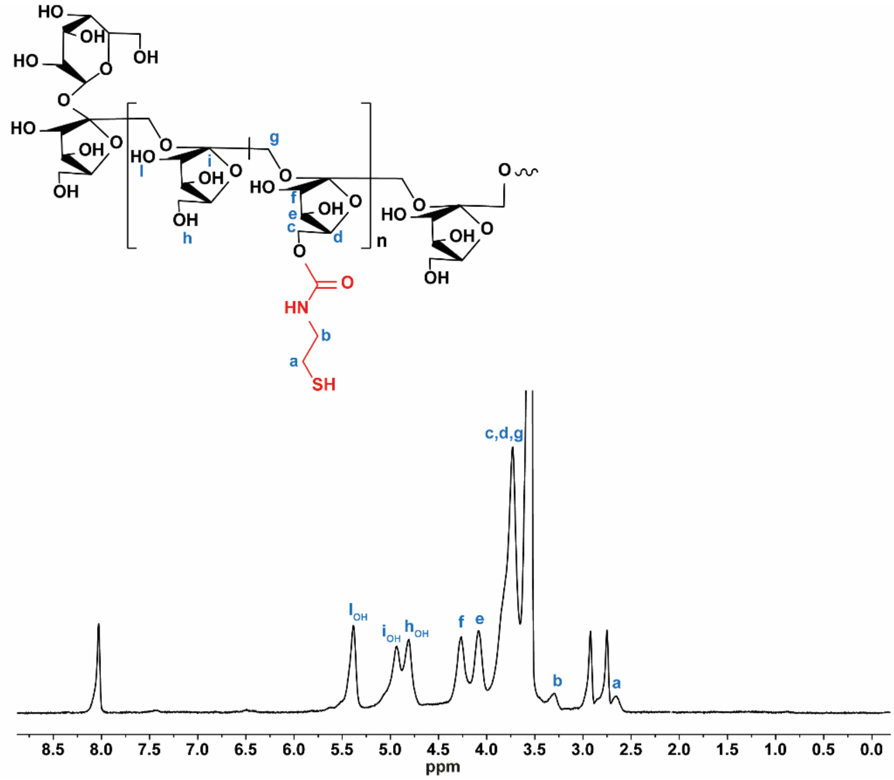

Figure S1. ^{1}H-NMR spectrum of intermediate Inu-Cys-SH.

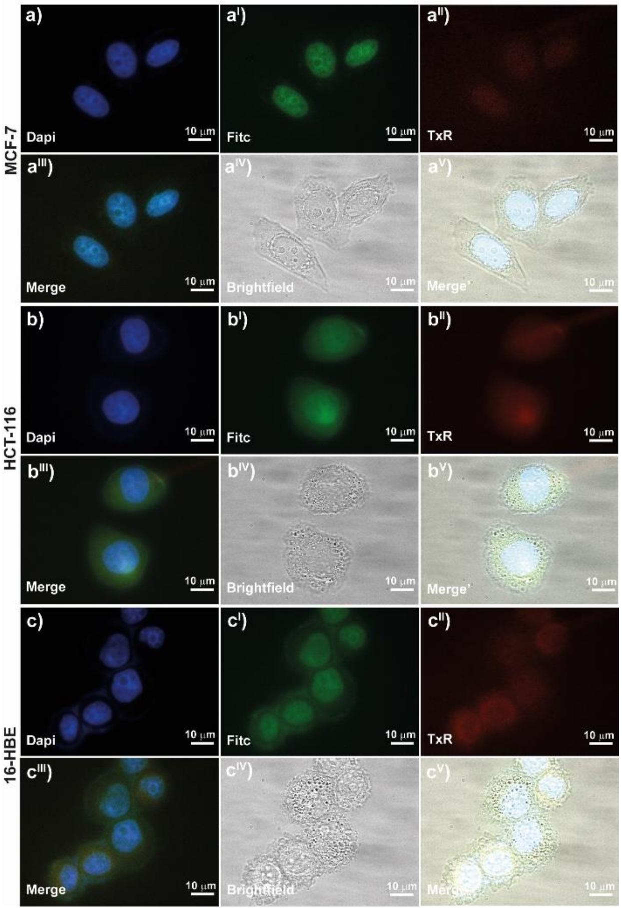

Figure S2. Cell uptake of loaded INU-Cys-TC@SN-38 on MCF-7 (a-a^{V}), HCT-116 (b-b^{V}) and 16-HBE (c-c^{V}) after 2 h of incubation.

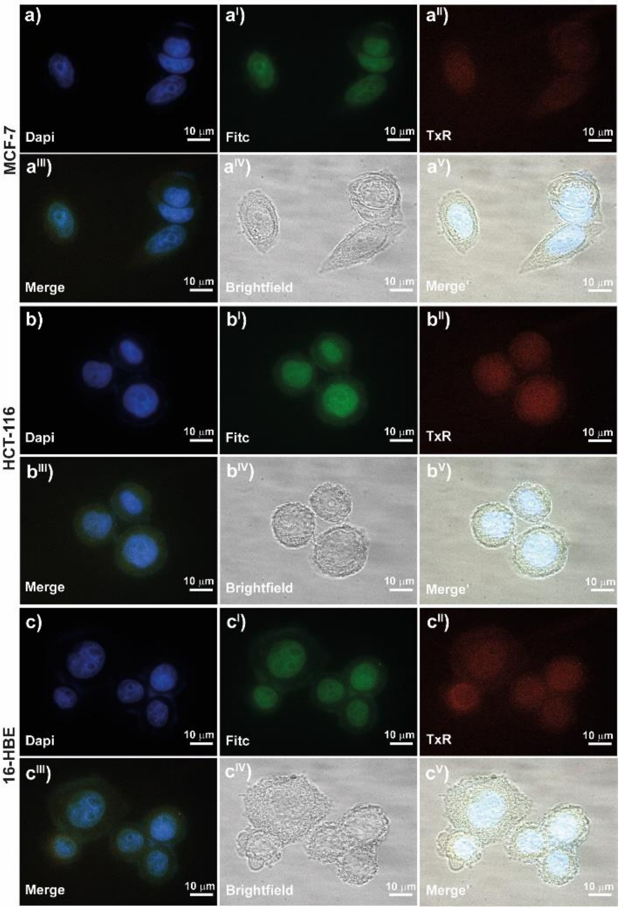

Figure S3. Cell uptake of loaded INU-Cys-TC@SN-38 on MCF-7 (a-a^{V}), HCT-116 (b-b^{V}) and 16-HBE (c-c^{V}) after 6 h of incubation.

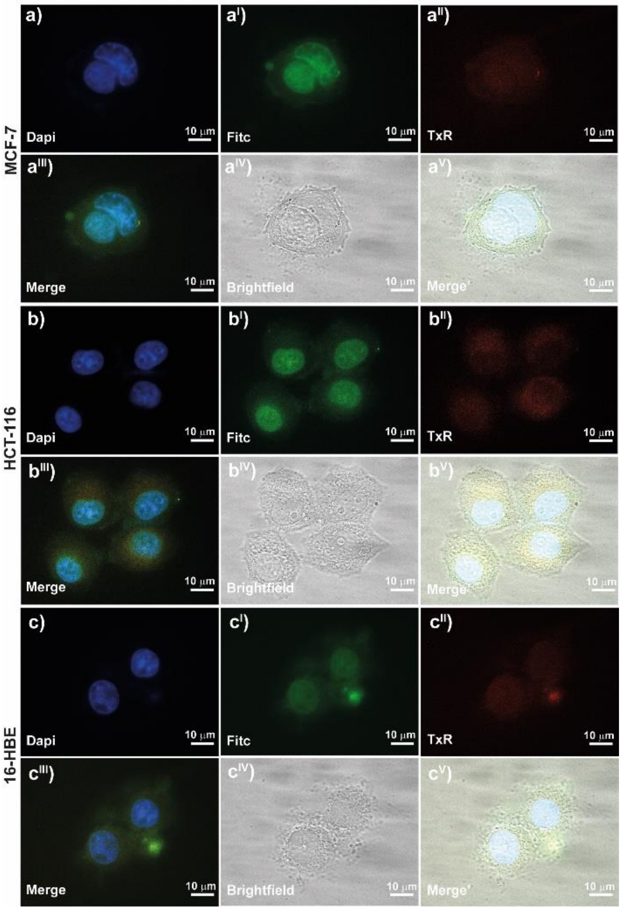

Figure S4. Cell uptake of loaded INU-Cys-TC@SN-38 on MCF-7 (a-a^{V}), HCT-116 (b-b^{V}) and 16-HBE (c-c^{V}) after 24 h of incubation.
